# Development of high-affinity nanobodies specific for Na_V_1.4 and Na_V_1.5 voltage-gated sodium channel isoforms

**DOI:** 10.1016/j.jbc.2022.101763

**Published:** 2022-02-21

**Authors:** Lakshmi Srinivasan, Vanina Alzogaray, Dakshnamurthy Selvakumar, Sara Nathan, Jesse B. Yoder, Katharine M. Wright, Sebastián Klinke, Justin N. Nwafor, María S. Labanda, Fernando A. Goldbaum, Arne Schön, Ernesto Freire, Gordon F. Tomaselli, L. Mario Amzel, Manu Ben-Johny, Sandra B. Gabelli

**Affiliations:** 1 Department of Biophysics and Biophysical Chemistry, The Johns Hopkins School of Medicine, Baltimore, Maryland, USA; 2 Fundación Instituto Leloir, IIBBA-CONICET, Buenos Aires, Argentina; 3 ForteBio, Sartorius BioAnalytical Instruments, Fremont, California, USA; 4 Department of Biology, The Johns Hopkins University Krieger School of Arts and Science, Baltimore, Maryland, USA; 5 Department of Medicine, The Johns Hopkins University School of Medicine, Baltimore, Maryland, USA; 6 Department of Physiology and Cellular Biophysics, Columbia University, New York, New York, USA; 7 Department of Oncology, The Johns Hopkins University School of Medicine, Baltimore, Maryland, USA

**Keywords:** nanobody, Na_V_1.4, Na_V_1.5, voltage-gated sodium channel, *Lama glama*, X-ray diffraction, biolayer interferometry, FRET, hIPSC-CM, Ab, antibody, BLI, biolayer interferometry, BSA, bovine serum albumin, CaM, calmodulin, cDNA, complementary DNA, CDR3, complementarity-determining region 3, CM, cardiomyocyte, CT, C-terminal region, dd, differentiation day, DMEM, Dulbecco's modified Eagle's medium, DSC, differential scanning calorimetry, FL, full length, GST, glutathione-S-transferase, HA, hemagglutinin, HEK293, human embryonic kidney 293 cell line, hiPSC, human-induced pluripotent stem cell, HRP, horseradish peroxidase, Na_V_, voltage-gated sodium channel, Nb, nanobody, Ni, nickel, NIH, National Institutes of Health, NTA, nickel–nitrilotriacetic acid, PBL, peripheral blood lymphocyte, PBST, PBS with Tween-20, PDB, Protein Data Bank, RT, room temperature, SEC, size-exclusion chromatography, T, truncated, TES, Tris–HCl, EDTA, and sucrose buffer, TN, Tris and NaCl buffer, VHH, variable domain of heavy chain of heavy-chain antibody

## Abstract

Voltage-gated sodium channels, Na_V_s, are responsible for the rapid rise of action potentials in excitable tissues. Na_V_ channel mutations have been implicated in several human genetic diseases, such as hypokalemic periodic paralysis, myotonia, and long-QT and Brugada syndromes. Here, we generated high-affinity anti-Na_V_ nanobodies (Nbs), Nb17 and Nb82, that recognize the Na_V_1.4 (skeletal muscle) and Na_V_1.5 (cardiac muscle) channel isoforms. These Nbs were raised in llama (*Lama glama*) and selected from a phage display library for high affinity to the C-terminal (CT) region of Na_V_1.4. The Nbs were expressed in *Escherichia coli*, purified, and biophysically characterized. Development of high-affinity Nbs specifically targeting a given human Na_V_ isoform has been challenging because they usually show undesired crossreactivity for different Na_V_ isoforms. Our results show, however, that Nb17 and Nb82 recognize the CTNa_V_1.4 or CTNa_V_1.5 over other CTNav isoforms. Kinetic experiments by biolayer interferometry determined that Nb17 and Nb82 bind to the CTNa_V_1.4 and CTNa_V_1.5 with high affinity (*K*_*D*_ ∼ 40–60 nM). In addition, as proof of concept, we show that Nb82 could detect Na_V_1.4 and Na_V_1.5 channels in mammalian cells and tissues by Western blot. Furthermore, human embryonic kidney cells expressing holo Na_V_1.5 channels demonstrated a robust FRET-binding efficiency for Nb17 and Nb82. Our work lays the foundation for developing Nbs as anti-Na_V_ reagents to capture Na_V_s from cell lysates and as molecular visualization agents for Na_V_s.

The nine human isoforms of voltage-gated sodium channels (Na_V_1.1–1.9) rapidly respond to changes in cellular membrane potential by allowing the passage of Na^+^ ions into the cell. They play an important role in the generation of the action potential in excitable tissues, such as skeletal muscle, heart, and nerves (1). Mutations in the cytoplasmic C-terminal (CT) region of these proteins have been implicated in human genetic diseases, such as generalized epilepsy with febrile seizures, hypokalemic periodic paralysis, myotonia, long-QT syndrome, and Brugada syndrome (2, 3, 4). Given the physiological importance of the Na_V_ isoforms in normal physiology and disease, development of reagents for their study, such as antibodies (Abs), has been an important part of Na_V_ research. Specifically targeting each individual isoform, however, has been challenging, because of their high sequence identity. To achieve tissue specificity and avoid off-target side effects of anti-Pan Na_V_ Abs, there is an increasing need for biologicals with high solubility, stability, and specificity (5, 6).

Nanobodies (Nbs) are single variable heavy-chain (VHH) immunoglobulin domains derived from heavy-chain–only Abs produced in camelids, such as camels, llamas (*Lama glama*), and alpacas. Nbs are small prolate-shaped molecules (∼15 kDa) that fully retain the epitope-recognizing function in a single-chain Ab. They may be selected to contain an extended and flexible complementarity-determining region 3 (CDR3) loop partly contributing to their high epitope affinity and their ability to better access smaller and cryptic epitopes (7). Moreover, VHH domains are amenable to cloning and protein modifications and can be produced in bacterial expression systems in scalable amounts. Nbs also display superior solubility, stability, *in vivo* half-lives, and pharmacodynamics compared with conventional Abs (8). For example, Nbs to P2x channel proteins have been shown to display greater therapeutic potential than conventional Abs for modulating channel function and reducing the *in vivo* inflammation caused by P2X7 (9, 10, 11, 12). Nbs have also been used as crystallization chaperones (7, 13, 14), visualization agents (15, 16, 17), *in vivo* radiotracers (18), pulldown baits (19, 20), intracellular pathway modulators (21), virus neutralization agents (22, 23), and therapeutics agents (24, 25, 26, 27). In this study, we have raised, selected, and characterized Nb clones that recognize the CT region of two Na_V_ isoforms: Na_V_1.4 (skeletal muscle) and Na_V_1.5 (cardiac muscle). We selected the CT domain to serve as an antigen for Nb production since the sequences are more divergent than in other regions of the channels such as the pore-forming regions. Furthermore, the CT domain of the channel is the binding site for channel-interacting proteins, which regulate their activity. This would potentially allow the selection of Nbs that are specific for a given isoform or to a particular state of the channel. Moreover, the available cryo-EM structures have not fully resolved details of these functionally relevant CTNa_V_ domains. Hence, Nbs targeted to the CT could potentially stabilize these regions, thereby aiding their structure elucidation. In this study, we detail the production, biophysical characterization, and unique structural features of two Nbs that recognize Na_V_1.4 and Na_V_1.5 channels and show results highlighting their potential to serve as molecular tools to study Na_V_ proteins *in vitro* as well as in cells and tissues.

## Results

### Generation and identification of Na_V_1.4-specific Nbs

**Figure 1:**
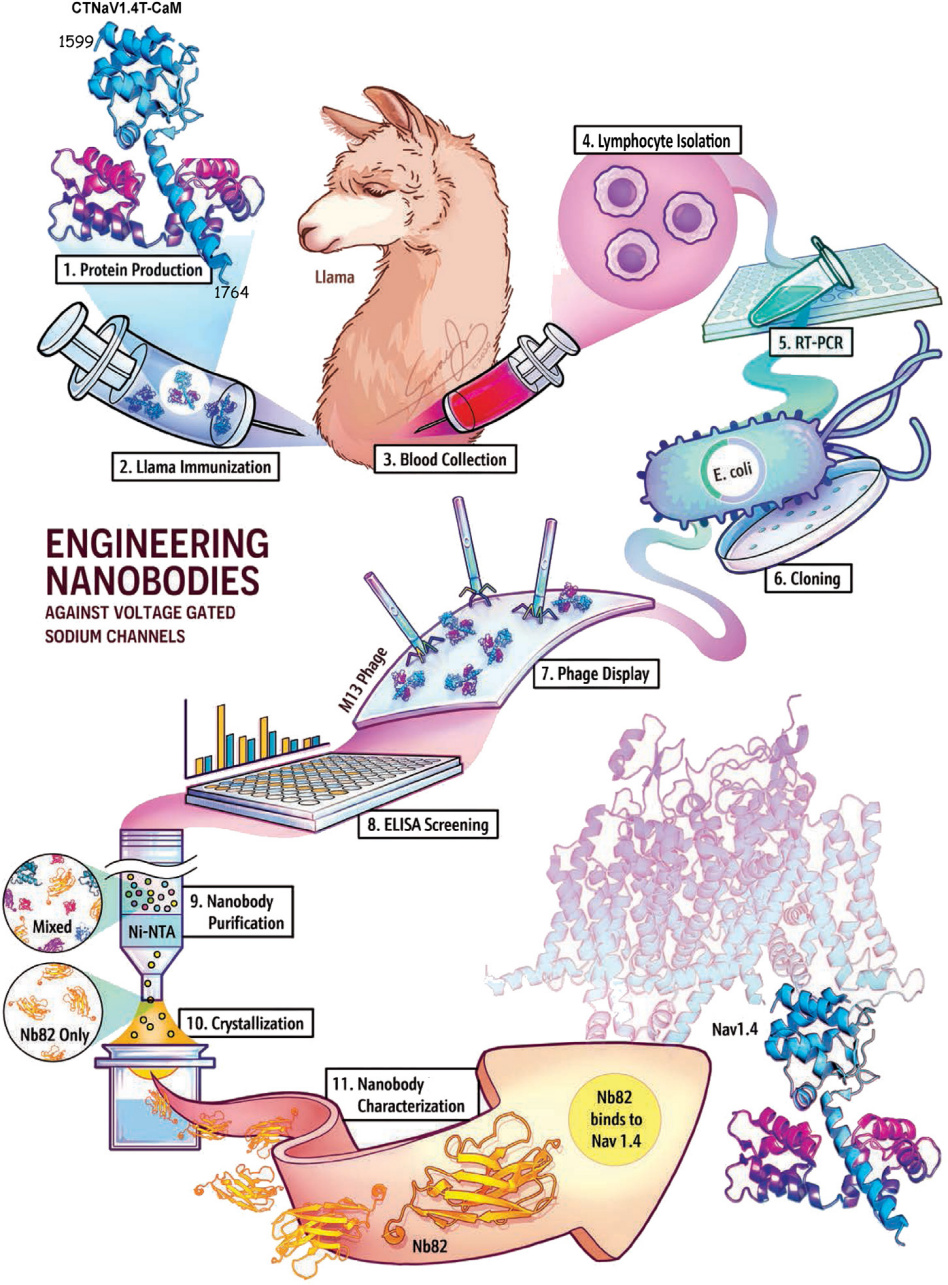
**Scheme describing the steps used for the selection of high-affinity nanobodies (Nbs) specific for voltage-gated sodium channels.** Step 1: Production of CTNa_V_1.4T–CaM to use as antigen. Step 2: Llama immunization with CTNa_V_1.4T–CaM. Step 3: Collection of immune sera from llama on day 35 and day 40 postimmunization. Step 4: Isolation of lymphocytes to extract total RNA. Step 5: RT–PCR to obtain complementary DNA of VHH domains. Step 6: Cloning of VHH complementary DNA (750 bp) into *Escherichia coli* shuttle vector for phage display. Step 7: Phage display for selection of Nbs against CTNa_V_1.4T–CaM in M13 phage. Step 8: Screening phage library by ELISA for high-binding Nb clones. Step 9: Cloning in pHEN6 vector and purification of selected Nb clones (Nb17, Nb30, and Nb82) in *E. coli*. Step 10: Crystallization of Nb82 using commercially available sparsematrix screens. Step 11: Characterization of Nb binding to Na_V_1.4 and Na_V_1.5 isoforms. This rendition was contributed by Sora Ji (Sorajistudio.com). CaM, calmodulin.

To facilitate the selection of Nbs with isoform specificity, we analyzed the homology amongst Na_V_ isoforms to choose a divergent region. Sequence homology analysis amongst the Na_V_ isoforms revealed that the linker between domains 1 and 2 (1–2 linker, amino acids 481–581 in Na_V_1.4) is the most divergent with pairwise identity between 40 and 55%, whereas the CTNa_V_ (1599–1836 amino acids in Na_V_1.4) domain displays pairwise identity of 60 to 70% (Table S1). The fact that CTNa_V_–calmodulin (CaM) is known to fold as a globular domain that can be purified and crystallized (28, 29) cemented the CTNa_V_ domain in complex with CaM (CTNa_V_–CaM) as the antigen of choice. To generate, select, and produce the Nbs (Fig. 1), a llama was immunized with purified CTNa_V_1.4T in complex with CaM (CTNa_V_1.4T–CaM, amino acids 1599–1764 CTNa_V_1.4T, Table S2 and Fig. 1, step 1 and 2), and its humoral immune response was evaluated by ELISA (Figs. S1*A* and 1, steps 3 and 4) (29). mRNA was extracted from isolated peripheral blood lymphocytes (PBLs; Fig. 1, step 4) and PCR amplified (Fig. 1, step 5). The VHH complementary DNA (cDNA) was cloned into a phagemid vector pHEN4 to obtain the VHH phage library (Fig. 1, step 6).

**Figure 2:**
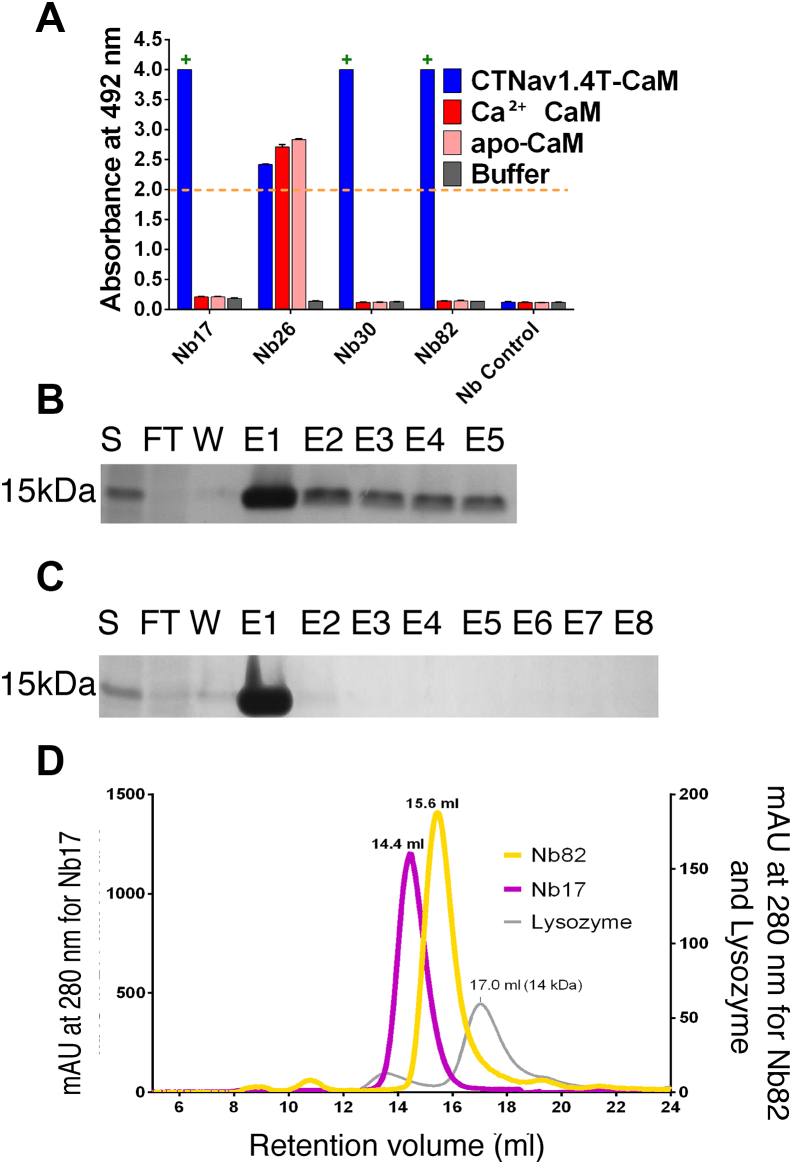
**Identification of nanobodies (Nbs) specific for Na**_**V**_**1.4.***A*, ELISA of the periplasmic extract using positively selected clones. Absorbances higher than 2 (*orange dashed line*) were considered positive. “+” signs over Nb17, Nb30, and Nb82 indicate that they are specific to CTNa_V_1.4T–CaM but not to Ca^2+^ CaM or apo-CaM. *B*, SDS-PAGE gel of IMAC purification of Nb17. *C*, same as (*B*) for Nb82. *D*, size-exclusion chromatogram of Nb17 (17.4 kDa, *purple*) and Nb82 (16.8 kDa, *yellow*). Lysozyme (14 kDa, *gray*) used as gel filtration molecular weight standard is also included. CaM, calmodulin; E, elution fraction; FT, flow-through; IMAC, immobilized metal affinity chromatography; S, supernatant; W, wash.

To identify Nbs that target CTNa_V_1.4–CaMs, we screened an Nb phage display library with an estimated complexity of >5.6 × 10^7^ clones (Fig. 1, step 7). Following two rounds of positive selection against CTNa_V_1.4T–CaM, a total of 87 randomly chosen clones were expressed and tested by ELISA (Fig. 1, step 8). Amongst them, 14 clones were found specific to CTNa_V_1.4–CaM (Fig. S1*B*). We further classified the 14 clones into four different families based on the length and variability of their CDR2 and CDR3 regions (Fig. S1*C*). One representative clone from each family was chosen and screened by periplasmic ELISA against CTNa_V_1.4T–CaM, Ca^2+^ CaM, and apoCaM proteins (Fig. 2*A*). Of these, Nb17, Nb30, and Nb82 were found to be specific to CTNa_V_1.4T–CaM (“+” signs) but not to Ca^2+^ CaM or apo-CaM. However, Nb26 recognized not only CTNa_V_1.4T–CaM but also Ca^2+^ CaM and apo-CaM. Based on this information, Nb17, Nb30, and Nb82 were chosen for expression and purification in *Escherichia coli* for further biophysical and biochemical characterization. The three selected Nb clones were subcloned into a pHEN6-His vector as a CT 6×-His tagged fusion protein and successfully expressed in the periplasm of *E. coli* Rosetta-gami 2(DE3) cells. The Nbs were extracted from the periplasm using a combination of thermal and osmotic shock methods (30). Of these, Nb30 was not pursued further because of its low expression levels. Nb17 and Nb82 were purified *via* nickel–nitrilotriacetic acid (Ni–NTA) chromatography, followed by size-exclusion chromatography (SEC; Figs.1, step 9 and 2, *B*–*D*). Both Nbs behave as monomers in solution and were detected as a single homogenous peak on SEC with retention volumes of 14.4 ml (Nb17) and 15.6 ml (Nb82) on a Superdex 75 10/300 GL column (Fig. 2*D*). These retention volumes are in agreement with those of Nbs reported in the literature (31, 32).

### Nb17 and Nb82 are thermally stable

**Figure 3:**
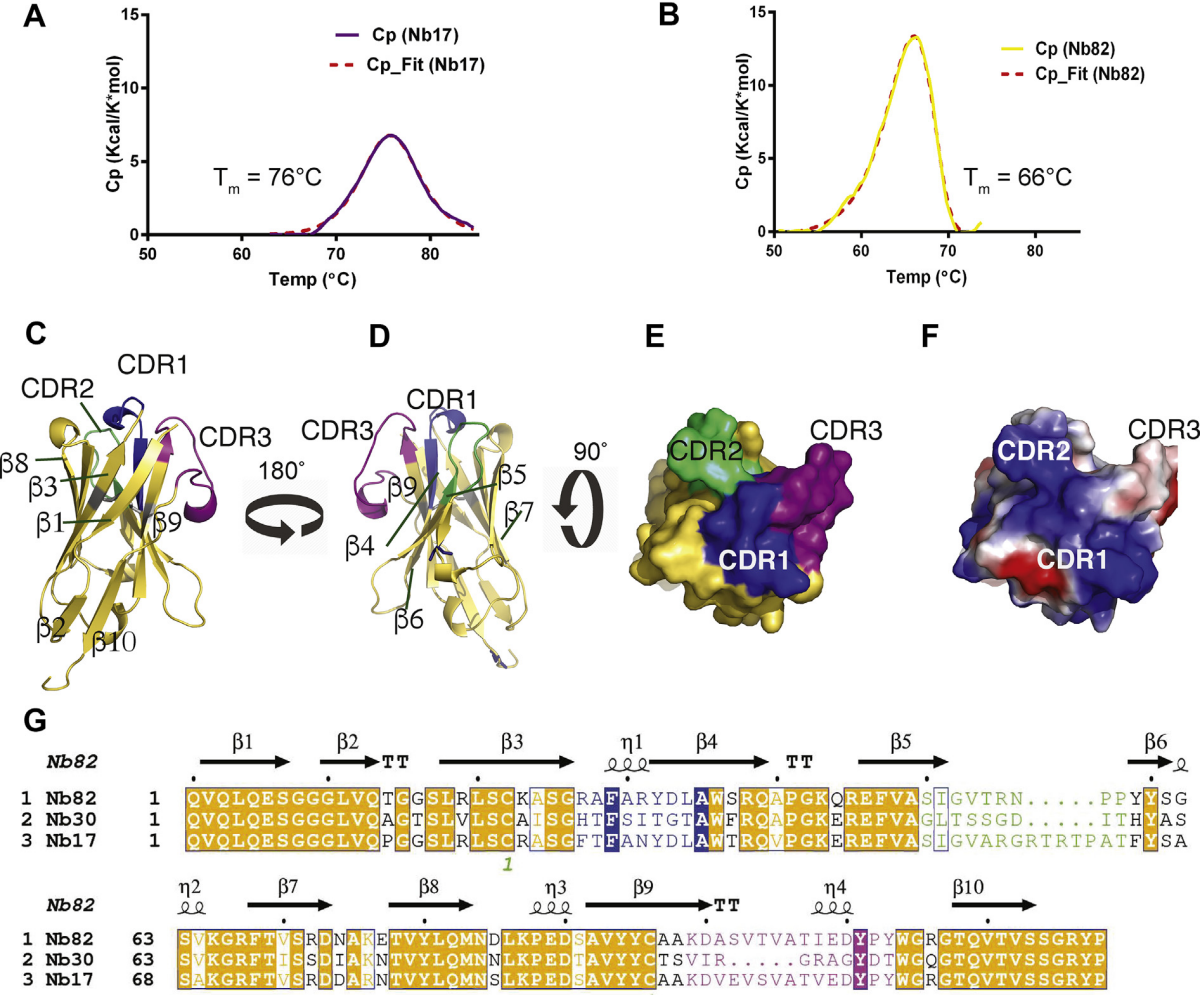
**Nanobody (Nb) thermal stability and crystal structure of Nb82.***A*, DSC curve showing the temperature denaturation of Nb17 undergoing reversible denaturation with *T*_m_ centered at 75.8 °C. *B*, same as *A*. Nb82 undergoes irreversible denaturation with *T*_m_ centered at 66.0 °C. *C*, cartoon representation of Nb82 (*yellow*) displaying the CDR1 (*blue*), CDR2 (*green*), and CDR3 (*magenta*). *D*, same as (*A*) with 180° rotation along the vertical axis. *E*, bird's eye view of Nb82 in (*A*) with surface coloring according to the CDRs. *F*, same as (*C*) with Nb82 surface colored according to the electrostatic charges. *G*, sequence alignment of Nb82, Nb30, and Nb17. The three CDR regions are color coded as CDR1 (*blue*), CDR2 (*green*), and CDR3 (*magenta*). The secondary structure elements of Nb82 are placed on top of the alignment. CDR, complementarity-determining region; DSC, differential scanning calorimetry.

The temperature stability of Nb17 and Nb82 was measured by differential scanning calorimetry (DSC). Nb17 is characterized by a *T*_m_ of 76 °C (Fig. 3*A*) and Nb82 by a *T*_m_ of 66 °C (Fig. 3*B*), suggesting both are relatively stable protein suitable for functional and structural characterization. Notably, Nb17 undergoes reversible temperature denaturation, whereas Nb82 undergoes irreversible denaturation. The different denaturation mechanisms probably originate from sequence differences. These different mechanisms are also reflected in the shape of the DSC curves (33). Also, Nb17 has a van’t Hoff enthalpy of 118 kcal/mol that is close to what is expected for a protein of this size undergoing two-state unfolding. For Nb82, the irreversible transition is kinetically controlled, with a noticeable change in shape of the curve, and characterized by an activation energy of 86 kcal/mol.

### Nb82 has an extended CDR3 loop

The structure of Nb82 was determined by X-ray crystallography to 2.0 Å resolution and refined to an *R*_work_/*R*_free_ of 0.19/0.24 with excellent geometry (Protein Data Bank [PDB] ID: 7R63; Table 1 and Fig. 3, *C*–*F*). The asymmetric unit includes four copies of Nb82 with almost identical conformations (Cα RMSD <0.30 Å, pairwise among all four chains, including 117 Cα carbons). Nb82 bears the classical immunoglobulin fold (Fig. 3, *C* and *D*) with two β-sheets of four antiparallel β-strands (β1–β3–β8–β7 and β6–β5–β4–β9) and a smaller β-sheet made up of two parallel β-strands, β2 and β10, that elongate the β6–β5–β4–β9 sheet. The structure displays good electron density for all portions of the four molecules (Table 1 and Fig. 3, *C* and *D*). The fold is decorated by three π-helices, two of which are in the epitope-recognizing CDR1 and CDR3, formed between the β3–β4 and β9–β10 loops, respectively. The third π-helix is on the loop connecting strands β3 and β4. CDR π-helices are a characteristic feature observed in other Nbs (14). CDR3, the usual major contributor for antigen recognition and specificity, folds as a random coil with a π-helix of seven residues that folds back onto β6–β5–β4–β9 sheet. Notably, the CDR1 and CDR2 present a positively charged patch (Fig. 3, *E* and *F*) suggesting that Nb82 favors an interaction with a large negatively charged surface on the Na_V_. Comparison of the sequence of Nb82 with Nb17 and Nb30 (Fig. 3*G*) and its structure (PDB ID: 7R63) with other Nbs available (PDB IDs: 5LMJ, 6H6Y, and 5LZ0 (14)) (Fig. S2) highlights the differences in the fold of their CDR3 (Fig. S2, *A* and *B*).

**Table 1:** Data collection and refinement statistics

Data collection	Nb82 (PDB: 7R63)
Space group	C2221
Cell dimensions	
*a*, *b*, *c* (Å)	77.7, 82.4, 170.7
*α*, *β*, *γ* (°)	90.00, 90.00, 90.00
Resolution (Å)^a^	29.67–2.0 (2.052–2.0)
*R*_merge_	0.07 (0.39)
*R*_pim_	0.030 (0.164)
CC1/2	0.999 (0.985)
⟨I/σ(I)⟩	14.5 (4.2)
Completeness (%)	99.9 (99.9)
Total reflections	249,354 (2845)
Unique reflections	37,476 (473)
Refinement	
*R*_work_/*R*_free_	0.19/0.24 (0.22/0.28)
No. of atoms	
Protein	4331
Ligand/ion	—
Water	278
*B*-factors (Å^2^)	
Protein	36.0
Water	44.6
RMSD	
Bond lengths (Å)	0.01
Bond angles (°)	1.57
Ramachandran plot	
Favored (%)	97.8
Allowed (%)	1.5
Disallowed (%)	0.7

a Values for the outer shell are given in parentheses.

### Nb17 and Nb82 specifically recognize CTNa_V_1.4 and CTNa_V_1.5 isoforms

**Figure 4:**
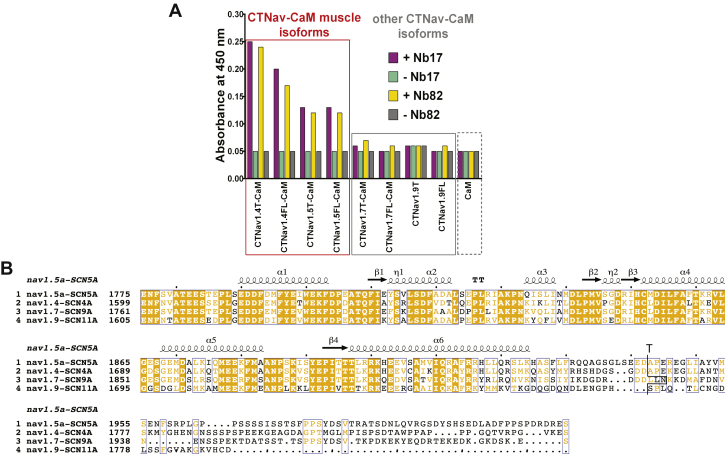
**Nb17 and Nb82 recognize the Na**_**V**_**-muscle isoforms.***A*, ELISA bar graphs using purified Nb17 (*magenta*), the absence of Nb17 (*green*), Nb82 (*orange–yellow*), and the absence of Nb82 (*gray*). The *red box* clusters Na_V_ proteins that represent muscle isoforms CTNa_V_1.4T–CaM, CTNa_V_1.4FL–CaM, CTNa_V_1.5T–CaM, and CTNa_V_1.5FL–CaM. The *gray box* clusters the other Na_V_ isoforms tested, CTNa_V_1.7T–CaM, CTNa_V_1.7FL–CaM, CTNa_V_1.9T, CTNa_V_1.9FL, and CaM. Data are representative of three independent experiments. *B*, sequence alignment of CTNa_V_1.4FL, CTNa_V_1.5FL, CTNa_V_1.7FL, and CTNa_V_1.9FL proteins. The *black trace* indicates the limits of the T CTNa_V_T–CaM constructs. CaM, calmodulin; FL, full length; Nb, nanobody; T, truncated.

To evaluate the specificity of the two Nbs, we performed ELISA experiments (Fig. 4*A*) with the purified Nbs and purified CTNa_V_ isoforms in complex with CaM (CTNa_V_1.4–CaM, CTNa_V_1.5–CaM, CTNa_V_1.7–CaM) and with CTNa_V_1.9, which does not form a complex with CaM. We used a truncated (T) or full-length (FL) construct of the CTNa_V_ isoforms (Fig. 4*B*). In ELISA experiments, Nb82 and Nb17 recognize both the T and FL versions of both CTNa_V_1.4–CaM (skeletal) and CTNa_V_1.5–CaM (cardiac). However, they do not recognize neuronal isoforms, CTNa_V_1.7–CaM or CTNa_V_1.9 (Fig. 4*A*). Importantly, they do not crossreact with free CaM (Fig. 4*A*).

**Figure 5:**
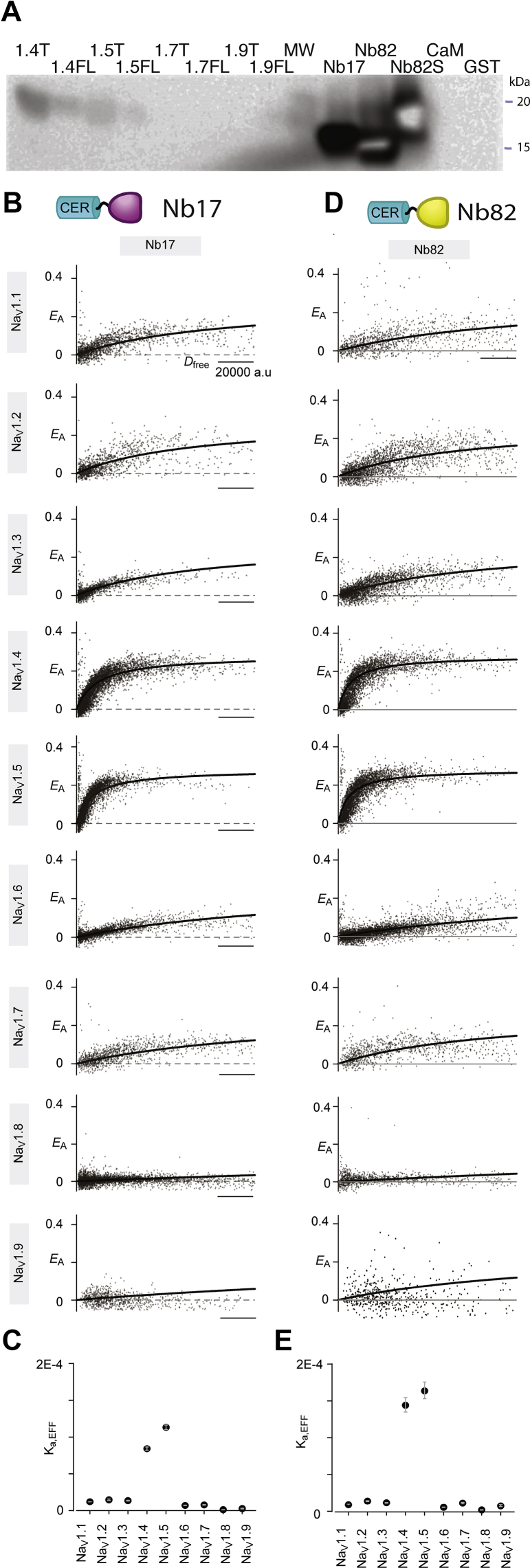
**Selectivity of Nb17 and Nb82 in detecting Na**_**V**_**1.4 and Na**_**V**_**1.5 channels.***A*, Western blot of purified CTNa_V_–CaM proteins, CaM alone, and Nb17-His, Nb82-His, CaM, and GST alone showing positive signals for CTNa_V_1.4T/FL (1.4T/1.4FL), CTNa_V_1.5T/FL (1.5T/1.5FL), and Nb17, Nb82-His and Nb82-StrepII (Nb82S). No signal is observed in the lanes that contained CTNa_V_1.7T/FL and CTNa_V_1.9T/FL suggesting that Nb82 does not recognize these two isoforms. Western blot was developed using Nb82-His as primary antibody and anti-HisHRP antibody as secondary. All Western blot data show one representative experiment of three. CTNa_V_–CaM proteins are labeled CTNa_V_1.xT (1.xT) and CTNa_V_1.xFL (1.xFL). *B*, Nbs tethered to Cerulean serve as a FRET donor, whereas Venus attached to amino-terminal region of CTNa_V_1.x serves as a FRET acceptor. Robust FRET is observed between Nb17 and CTNa_V_1.4/5. Other CTNa_V_s demonstrate reduced binding. FRET efficiency (*E*_A_) is plotted against the free donor concentration (*D*_free_). Each cell represents data from a single cell. *C*, the relative association constant, *K*_a,EFF_ (in arbitrary units) computed as 1/Kdeff from the fits in (*A*), demonstrates the preference of Nb17 for CTNa_V_1.4/1.5 over other Na_V_ isoforms. *D*, analysis of Nb82 shows strong FRET with CTNa_V_1.4/5. Other Venus-CTNa_V_1.x isoforms exhibit weaker binding. *E*, the *K*_a,EFF_ values confirm strong preference of Nb82 for CTNa_V_1.4/5 over other CTNa_V_1.x isoforms. CaM, calmodulin; FL, full length; GST, glutathione-*S*-transferase; HRP, horseradish peroxidase; Nb, nanobody; T, truncated.

Consistent with these findings, Western blot analysis demonstrated that His-tagged Nb82 as a primary Ab detected purified CTNa_V_1.4T, CTNa_V_1.4FL, CTNa_V_1.5T, and CTNa_V_1.5FL, resulting in signals corresponding to their respective molecular weights (Fig. 5*A*). On the other hand, no signal was detected for purified CTNa_V_1.7, CTNa_V_1.9, CaM alone, and glutathione-*S*-transferase (GST) (Fig. 5*A*), suggesting that Nb82 is specific to Na_V_1.4 and Na_V_1.5 channels.

To fully establish the selectivity of Nb17 and Nb82, we utilized a flow cytometry–based FRET two-hybrid assay to systematically probe the interaction of the two Nbs with CTNa_V_1.1–CTNa_V_1.9. We engineered Nb17 and Nb82 with a Cerulean fluorescent protein as a FRET donor and the CT domains of Na_V_1.x with the Venus fluorescent protein, as FRET acceptor. Stochastic expression of the FRET pairs resulted in variable FRET efficiencies (*E*_A_) in individual cells. A saturating binding relation was then constructed by correlating *E*_A_ with the free donor concentration (*D*_free_). Nb82 and Nb17 exhibited robust FRET with CTNa_V_1.4 and CTNa_V_1.5, whereas other isoforms showed qualitatively weaker FRET (Fig. 5, *B* and *D*). To quantify further, we calculated a relative association constant, *K*_a,EFF_ (in arbitrary units) as 1/*K*_d,EFF_ (the relative dissociation constant) from the fits in panels *B* and *D*. Indeed, both Nb17 and Nb82 demonstrated 7-fold to 100-fold higher *K*_a,EFF_ for Na_V_1.4 and Na_V_1.5 as compared with other Na_V_ isoforms (Fig. 5, *C* and *E*). Taken together, these results highlight the exquisite selectivity of Nb17 and Nb82 for the muscle Na_V_ channels.

**Figure 6:**
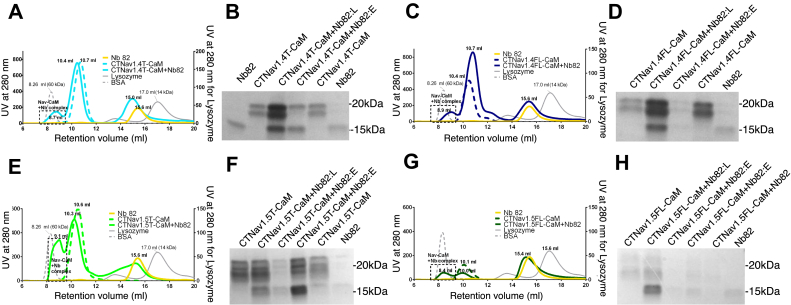
**Nb82 forms a complex with CTNa**_**V**_**1.4–CaM and CTNa**_**V**_**1.5–CaM detected by size-exclusion chromatography (SEC).***A*, SEC profile for CTNa_V_1.4T–CaM + Nb82 (*solid blue line*) showing the appearance a new peak to the left of the CTNa_V_1.4T–CaM peak (*dashed line*) indicating complex formation. *B*, SDS-PAGE gel showing elution fractions from (*A*). The CTNa_V_1.4T–CaM + Nb82 complex elutes at 8.7 ml. *C* and *D*, same as *A* and *B*, using construct CTNa_V_1.4FL–CaM. The CTNa_V_1.4FL–CaM + Nb82 elutes at 8.9 ml. *E*, SEC profile for CTNa_V_1.5T–CaM + Nb82 (*solid green line*) showing the appearance of the peak of the complex to the left at 9.1 ml compared with CTNa_V_1.5T–CaM (*dashed line*) at 10.6 ml. *F*, SDS-PAGE gel showing elution fractions from (*E*). *G* and *H*, same as (*E*) and (*F*) using construct CTNa_V_1.5FL–CaM. The CTNa_V_1.5FL–CaM + Nb82 complex elutes at 8.4 ml. Gel filtration molecular weight standards. BSA (66 kDa, *dashed gray line*) and lysozyme (14 kDa, *solid gray line*) are included in *A*, *C*, *E*, and *G*. BSA, bovine serum albumin; CaM, calmodulin; FL, full length; Nb, nanobody; T, truncated.

Thus affirmed, we further characterized the CTNa_V_–CaM and Nb82 interactions by analyzing the CTNa_V_X–CaM + Nb mixtures (where X equals 1.4 or 1.5) by SEC (Fig. 6). In the profiles, we observed that the CTNa_V_1.4T–CaM + Nb82 (Fig. 6, *A* and *B*) and CTNa_V_1.4FL–CaM + Nb82 complexes (Fig. 6, *C* and *D*) elute about 2 ml earlier than the equivalent complexes in the absence of Nb82. SDS-PAGE gels of the elution fractions show that the new peaks contain all three proteins; CTNa_V_1.4, CaM, and the Nb82 (Fig. 6, *B* and *D*). Also, the complexes elute at retention volumes that correspond to the molecular weight of the CTNa_V_1.4–CaM + Nb complex 1:1 stoichiometry. CTNa_V_1.5 showed a similar behavior. The complexes CTNa_V_1.5T–CaM + Nb82 and CTNa_V_1.5FL–CaM + Nb82 elute 1.0 and 1.5 ml before the CTNa_V_1.5T–CaM complex, respectively (Fig. 6, *E* and *G*). The SDS-PAGE gels of the peak fractions confirm the presence of all three proteins (CTNa_V_1.5, CaM, and Nb) (Fig. 6, *F* and *H*).

The mixture of CTNa_V_1.5–CaM + Nb17 on SEC does not show a fully resolved new peak, typical of the formation of a complex. However, in the CTNa_V_1.5T–CaM + Nb17 (Fig. S3, *A* and *B*) and CTNa_V_1.5FL–CaM + Nb17 (Fig. S3, *C* and *D*), peaks elute 0.5 ml before the CTNa_V_–CaMs without the Nb followed by an asymmetric Nb17 peak. Also, in the SDS-PAGE gels of the elution fractions, the peaks corresponding to the CT Na_V_1.5–CaM + Nb17 complex shows the presence of all three proteins; CTNa_V_1.5, CaM, and Nb17. These results imply that both Nb17 and Nb82 interact with the CTNa_V_–CaMs but affect their hydrodynamic radii differently.

### Nbs bind with nanomolar affinities to Na_V_1.4 and Na_V_1.5

**Figure 7:**
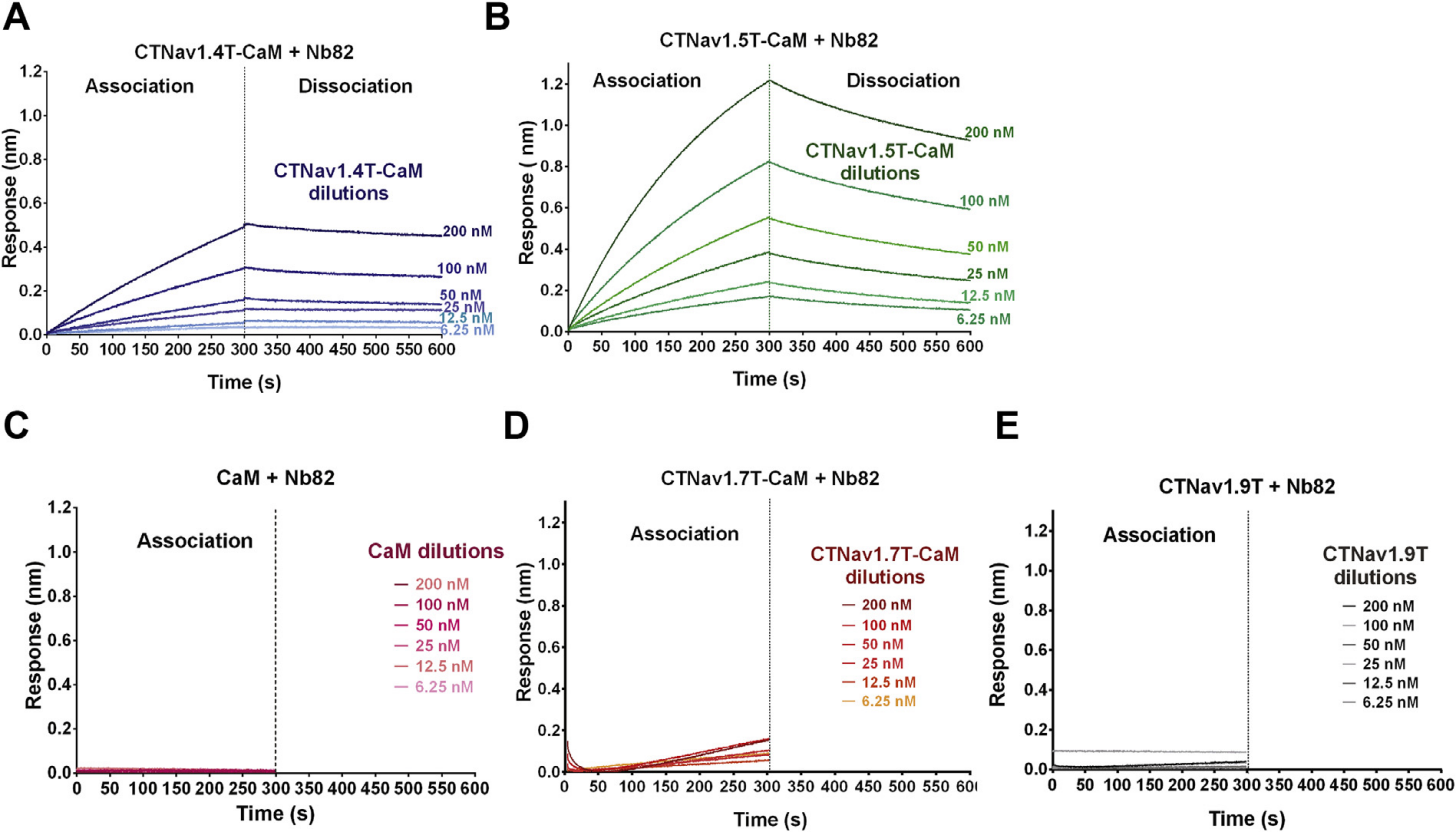
**Nb82 binds to CTNa**_**V**_**1.4T–CaM and CTNa**_**V**_**1.5T–CaM with nanomolar affinity.***A*, BLI sensorgram of Nb82 titrated with CTNa_V_1.4T–CaM at concentrations 6.25, 12.5, 25, 50, 100, and 200 nM plotted as nanometer shift with time. *B*, same sensorgram as *A* but for Nb82 titrated with CTNa_V_1.5T–CaM. *C*–*E*, sensorgrams showing no binding of Nb82 to CaM alone, CTNa_V_1.7T–CaM, or CTNa_V_1.9T, respectively. BLI, biolayer interferometry; CaM, calmodulin; Nb, nanobody; T, truncated.

**Figure 8:**
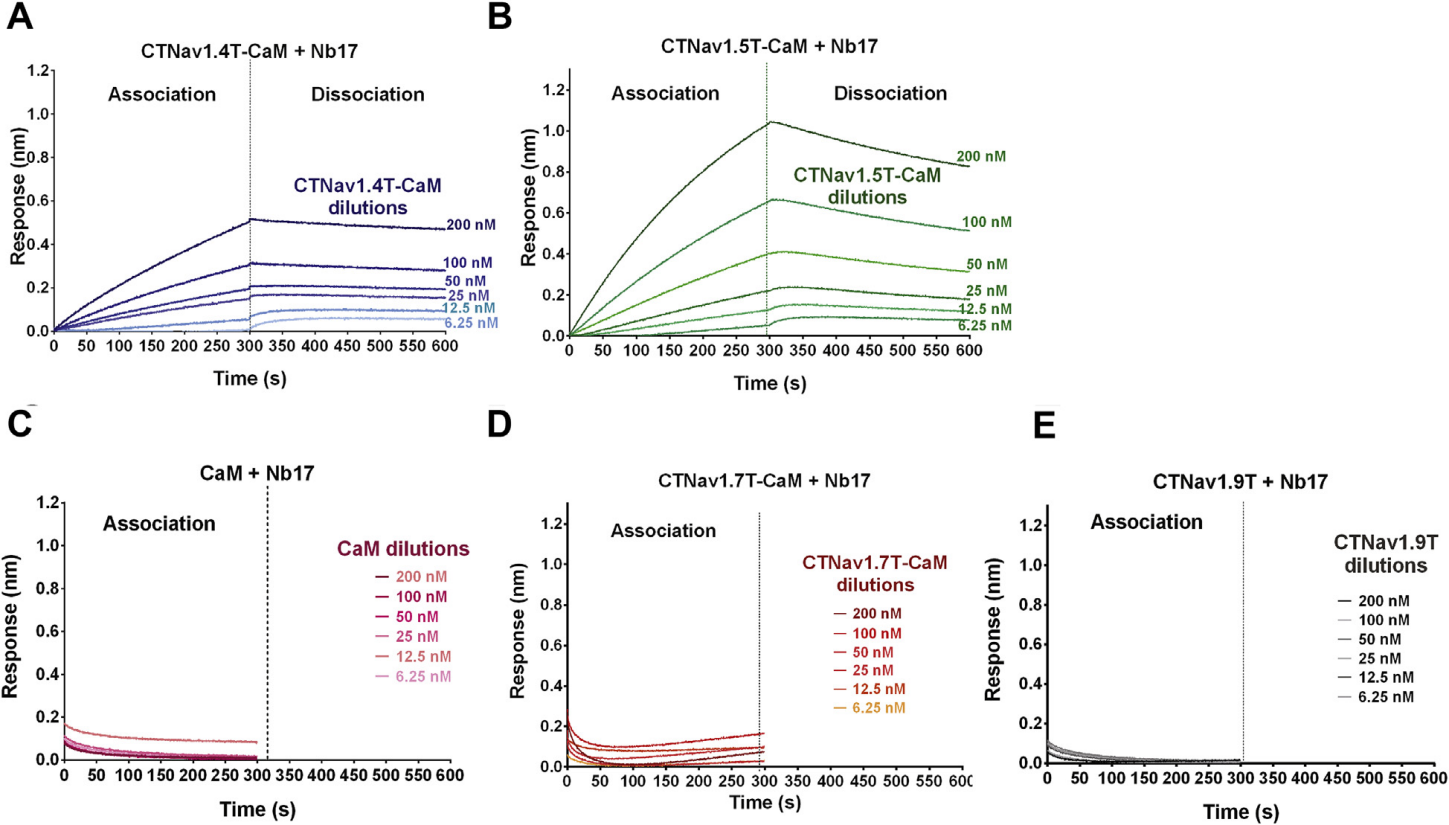
**Nb17 binds to CTNa**_**V**_**1.4T–CaM and CTNa**_**V**_**1.5T–CaM with nanomolar affinity.***A*, BLI sensorgram of Nb17 titrated with CTNa_V_1.4T–CaM at concentrations 6.25, 12.5, 25, 50, 100, and 200 nM plotted as nanometer shift with time. *B*, same sensorgram as (*A*) but for Nb17 titrated with CTNa_V_1.5T–CaM. *C*–*E*, sensorgrams showing no binding of Nb17 to CaM alone, CTNa_V_1.7T–CaM, or CTNa_V_1.9T, respectively. BLI, biolayer interferometry; CaM, calmodulin; Nb, nanobody; T, truncated.

We further probed the molecular interactions of the CTNa_V_1.4–CaM + Nb and CTNa_V_1.5–CaM + Nb ([CTNa_V_X]–CaM + Nb; X = 1.4 or 1.5) complexes by determining the kinetic parameters of binding using biolayer interferometry (BLI) (Figs. 7 and 8). The change in resonance with time was recorded at different concentrations of purified CTNa_V_1.XT–CaM proteins or isolated CaM. A 1:1 binding of the Nbs to CTNa_V_XT–CaM proteins was observed with signals reaching steady state in 300 s. Analysis of the BLI dose responses of association and dissociation curves indicates that Nb82 binds to CTNa_V_1.4T–CaM and CTNa_V_1.5T–CaM with affinities of 50.2 ± 0.1 and 63.2 ± 0.07 nM, respectively (Fig. 7, *A* and *B* and Table 2). However, no binding to Nb82 was observed when isolated CaM was used as an analyte (Fig. 7*C*) evidenced by a nonassociation/no-binding curve. In addition, as estimated from ELISA results, no binding to Nb82 was observed when CTNa_V_1.7T–CaM or CTNa_V_1.9T were used as analytes (Fig. 7, *D* and *E*). Similarly, with Nb17 (Fig. 8, *A* and *B*), BLI experiments show that it binds to CTNa_V_1.4T–CaM and CTNa_V_1.5T–CaM with dissociation constants (*K*_*D*_s) of 41.0 ± 0.1 nM and 60.5 ± 0.06 nM, respectively (Table 2 and Fig. 8*C*) but not to isolated CaM. Moreover, as expected, no binding to Nb17 was observed when CTNa_V_1.7T–CaM or CTNa_V_1.9T was used as analytes (Fig. 8, *D* and *E*). In these experiments, the lack of binding of Nb17 and Nb82 to isolated CaM and to CTNa_V_1.7–CaM and CTNa_V_1.9 indicates that the epitope for Nb82 binding is on the Na_V_1.4(5) channels or on the Na_V_1.4(5)–CaM interface in an area different enough from that of Na_V_17 or Na_V_1.9.

**Table 2:** Kinetic and binding parameters determined by BLI for CTNa_V_1.4T–CaM and CTNa_V_1.5T–CaM titrated with Nb17 and Nb82

Nb (ligand)	Na_V_ proteins (analyte)	*K*_*D*_ (nM)	*k*_on_ (M^−1^ s^−1^)	*k*_dis_ (1/s)
Nb17	CTNa_V_1.4T–CaM	41.1 ± 9.89	1.80E^04^ ± 3.50E^02^	7.39E^−04^ ± 1.05E^−05^
	CTNa_V_1.5T–CaM	60.5 ± 5.80	1.78E^04^ ± 1.51E^02^	1.07E^−03^ ± 4.74E^−06^
Nb82	CTNa_V_1.4T–CaM	50.2 ± 8.87	8.98E^03^ ± 1.32E^02^	4.51E^−04^ ± 4.41E^−06^
	CTNa_V_1.5T–CaM	63.2 ± 6.75	1.78E^04^ ± 1.71E^02^	1.13E^−03^ ± 5.28E^−06^

### Nb17 and Nb82 detect Na_V_1.5 channel in live cells and tissue homogenates

**Figure 9:**
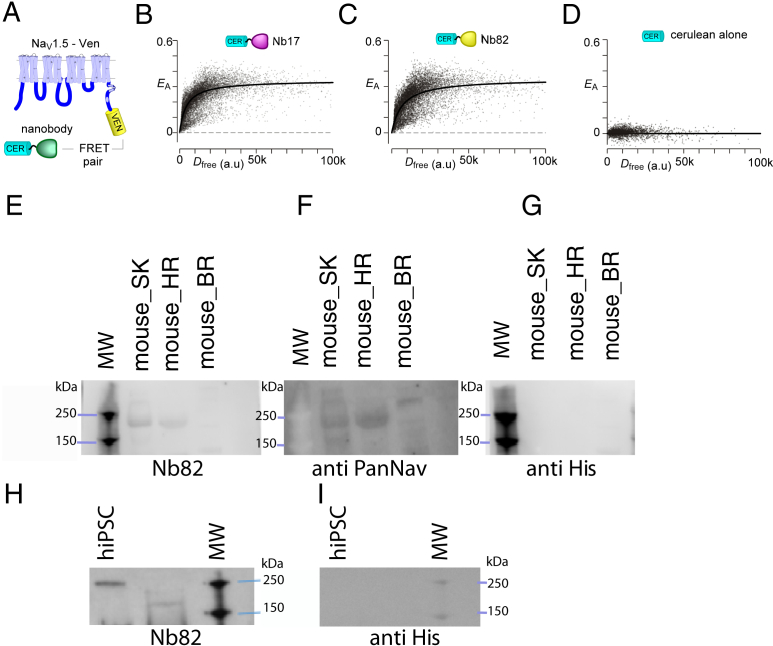
**Nanobodies (Nbs) as tools to detect Na**_**V**_**channels from live cells and tissue homogenates.***A*, schematic of FRET two-hybrid assay to probe live-cell binding of Nbs to holo-Na_V_1.5 channels. Nbs tethered to Cerulean serve as a FRET donor, whereas Venus attached to Na_V_1.5 serves as a FRET acceptor. *B*, robust FRET is observed between Nb17 and Na_V_1.5. FRET efficiency (*E*_A_) is plotted against the free donor concentration (*D*_free_). Each cell represents data from a single cell. *C*, analysis of Nb82 also shows strong FRET with Na_V_1.5. *D*, no appreciable FRET is observed between Na_V_1.5-Venus and Cerulean alone. *E*, Western blot showing Nb82-His used as the primary antibody recognizing Na_V_1.4(5) channels from tissues; mouse skeletal muscle, mouse heart, and brain. Blot developed using an anti-His-HRP antibody. *F*, same as (*E*) developed using the Pan-Na_V_ antibody (Sigma). *G*, same as (*E*) and (*F*) developed using only anti-His HRP antibody as a control. *H*, Western blot showing Nb82-His used as the primary antibody recognizing Nav1.5 from hiPSC–CMs. *I*, same as (*H*) developed using anti-His HRP antibody as a control. CM, cardiomyocyte; hiPSC, human-induced pluripotent stem cell; HRP, horseradish peroxidase.

To determine whether Nb17 and Nb82 interact with holo-Na_V_1.5 channels in live cells, we utilized flow cytometry–based FRET two-hybrid assay (34). To do so, we utilized Cerulean-tagged Nb17 and Nb82 as FRET donor, and we attached a Venus tag to the carboxy terminus of the FL Na_V_1.5 (FRET acceptor) (Fig. 9*A*) and obtained fluorescence measurements using a flow cytometer. Stochastic expression of the FRET pairs resulted in variable FRET efficiencies (*E*_A_) in individual cells. A saturating binding relation was then constructed by correlating *E*_A_ with the free donor concentration (*D*_free_). We observed robust FRET for both Nb17 (Fig. 9*B*) and Nb82 (Fig. 9*C*) with Na_V_1.5 confirming baseline association of the heterologously expressed Nb in live cells. By contrast, coexpression of Cerulean alone with Na_V_1.5 did not show appreciable FRET (Fig. 9*D*). These findings demonstrate the robust interaction of Nb17 and Nb82 with Na_V_1.5 in live cells, thus furnishing a new avenue to probe and manipulate Na_V_ channels in physiology.

### Nb82 as a Na_V_1.4 and Na_V_1.5 molecular detection reagent

To take advantage of the high affinity of the Nbs for CTNa_V_1.4 and CTNa_V_1.5, we used Nb82 as a molecular reagent to detect Na_V_s in cell and tissue lysates by Western blot. His-tagged Nb82 detects Na_V_1.4 in mouse skeletal muscle homogenates in addition to Na_V_1.5 in mouse heart homogenates as shown by a band at ∼220 kDa. Notably, Nb82 does not detect Na_V_s in brain tissue (Fig. 9*E*). The Western blot signal detected by the His-tagged Nb82 (Fig. 9*E*) is comparable to that detected by a commercially available anti-Pan Na_V_ Ab (Fig. 9*F*) as observed by signals corresponding to the same molecular weight for skeletal muscle and heart tissue samples in both blots. Moreover, the anti-Pan Na_v_ Ab detects a band at ∼250 kDa in mouse brain tissue (Fig. 9*F*) that is not observed with the Nb82 detection. Also, Nb82 detects Nav1.5 from human induced pluripotent stem cell–derived cardiomyocytes (hiPSC–CMs) differentiated from normal hiPSCs as observed by a band at ∼220 kDa (Fig. 9*H*). As a control, when the blots were reprobed with simply anti-His horseradish peroxidase (HRP) Ab (Fig. 9,*G* and *I*), no bands were observed at 220 to 250 kDa showing the specificity of detection by Nb82 as expected.

## Discussion

This study describes the generation and characterization of two Nbs, Nb17 and Nb82, that recognize and bind to the voltage-gated sodium channel Na_V_1.4 and Na_V_1.5 isoforms. The Nbs do not recognize isolated CaM, Na_V_1.7, or Na_V_1.9 channels showing their specificity for the Na_V_ muscle isoform, Na_V_1.4(5). Both Nbs have structural stabilities similar to those observed for other globular proteins. According to the survey of Robertson and Murphy (35), the average *T*_m_ for globular proteins is approximately 68 °C compared with the *T*_m_s of 76 and 66 °C for Nb17 and Nb82, respectively. Also, according to the survey of Robertson and Murphy, the mean enthalpy for a protein of 15 kDa at 70 °C is 115 kcal/mol, which is close to the van’t Hoff enthalpy of Nb17 suggesting structuring interactions similar to those observed for other proteins. The denaturation transition of Nb82 is irreversible at pH 8.0, which is not unseen for other proteins (36) that nevertheless undergo reversible denaturation at lower pH values.

The crystal structure of Nb82 reveals a long (15 amino acids) CDR3 for a llama-derived Nb that could possibly underlie the nanomolar binding affinities observed for the Nbs to Na_V_1.4 and Na_V_1.5.

Nb82 promises to be a high-affinity Na_V_1.4(5) protein detection reagent. Our SEC experiments demonstrate formation of stable CTNa_V_1.4(5)–CaM + Nb complexes. Importantly, binding kinetic experiments by BLI show nanomolar binding affinities for the Nbs to CTNa_V_1.4–CaM and CTNa_V_1.5–CaM proteins and no binding to CaM or CTNa_V_1.7–CaM and CTNa_V_1.9 proteins. Furthermore, flow-cytometric FRET measurements in human embryonic kidney 293 (HEK293) cells expressing FL Na_V_1.5 channel display high efficiency of binding of Nb17 and Nb82 to Na_V_1.5 channels on the cell surface but no binding to CaM-expressing cells. In HEK293 cells expressing CTNa_V_1.4 or CTNa_V_1.5 isoforms, flow-cytometric FRET measurements show binding to Nb17 or Nb82. In contrast, cells expressing any of the other CTNa_V_1.x isoforms displayed negligible binding. We further demonstrate that Nb82 can be used as a molecular reagent to detect Na_V_1.4(5) proteins in various types of cell and tissue lysates since it recognizes endogenous Na_V_1.4 channel from mammalian skeletal muscle in addition to Na_V_1.5 from mammalian heart and hiPSC–CMs.

Nbs have been selected for other voltage-gated channel–related subunits. For instance, the auxillary subunit of Ca_V_ channel, Ca_v_β, has been targeted to design a potent genetically encoded high-voltage–activated calcium channel inhibitor (37). It is worth highlighting that the anti-Na_V_1.4 and anti-Na_V_1.5 Nbs produced as part of this study carry a simple CT 6×Histidine tag, ensuring ease of production and scale up of anti-Na_V_ Nbs to serve as Na_V_ detection reagents, crystallization chaperones, and as *in vitro* and *in vivo* research tools and molecular probes.

## Experimental procedures

### Methods

#### CT Na_V_1.4–CaM protein expression for llama immunization

The GST-tagged CT region of Na_V_1.4 (amino acids 1599–1764), in complex with CaM (CTNa_V_1.4T–CaM), was expressed and purified from BL21-CodonPlus RIL *E. coli* cells using a GST-sepharose. Reduced l-glutathione eluted fractions were proteolyzed with PreScission protease followed by anion exchange chromatography and a final gel filtration chromatography as described by Yoder *et al*. (29). Purified CTNa_V_1.4T–CaM at 56 mg/ml was used for generation of single-chain Abs by llama immunization.

#### Llama immunization and construction of Nb library

The immunization protocol and the construction of the library were done as previously described (30, 38). In brief, llama was immunized three times intramuscularly every 15 days with 100 μg of purified CTNa_V_1.4T–CaM emulsified with complete Freund’s adjuvant. The humoral immune response in the sera was monitored by ELISA performed in plates coated with CTNa_V_1.4T–CaM. Forty-five days after the first immunization (D45), the animal was bled. PBLs were isolated from 300 ml of blood by Ficoll–Paque gradient centrifugation. Total RNA was purified from these cells and subjected to cDNA synthesis. The Nb coding regions were amplified by PCR using specific primers: forward (fwd_001): 5′-GTCCTGGCTGCTCTT CTACAAGG-3′; reverse (rvd_002): 5′-GGTACGTGCTGTTGAACTGTTCC-3′. The amplicons were purified from agarose gels, digested with PstI and NotI, and cloned into the pHEN4 phagemid vector downstream of the PelB-leader peptide and upstream of the hemagglutinin (HA) tag. *E. coli* TG1 cells were transformed with this vector for obtaining a phage display library. About 15 clones randomly chosen from the library were used for plasmid DNA preparation and run on agarose gel electrophoresis for qualitative analysis, where ˃70% of clones were found to be positive for Nbs.

#### Phage display selection of Na_V_1.4–CaM-specific Nbs

The panning was performed in MaxiSorp plates. Briefly, wells were sensitized with either 1 μg of CTNa_V_1.4T–CaM + 1 mM DTT or uncoated to serve as negative controls. After blocking with 3% skimmed milk in PBS, 1 × 10^12^ phages in PBS were added to each test and control coated wells and incubated for 2 h at room temperature (RT). Wells were then extensively washed with 25 mM Tris, 150 mM NaCl, 1 mM DTT, 0.05% Tween-20, pH 8.0, and bound phages were eluted with 0.25 mg/ml trypsin. Eluted phages were titrated and subjected to rounds of panning following the same procedure. Output phage titers were estimated by infection of TG1 cells and plating them on LB with 100 μg/ml ampicillin and 2% glucose. A total of 87 randomly chosen clones were grown in deep-well plates containing 1 ml of 2× TY (Tryptone 16 g, yeast extract 10 g, NaCl 5 g per L) medium added with 100 μg/ml ampicillin and 0.1% glucose for 3 h at 37 °C and 200 rpm until cell growth reached the exponential phase. The expression of Nbs was induced by adding 1 mM IPTG per well with shaking at 200 rpm for 4 h at 28 °C. The Nbs were obtained from the periplasm and tested by ELISA on plates sensitized with 1 μg of CTNa_V_1.4T–CaM. After washing with 25 mM Tris, 150 mM NaCl, 1 mM DTT, 0.05% Tween-20, pH 8.0, Nbs were detected by incubation with anti-HA Ab and developed using antirat IgG peroxidase conjugate. About 14 positive clones were selected for sequencing using universal M13 reverse as primer and then classified in families based on the length and variability of their CDR2 and CDR3. Multiple sequence alignments were done with Clustal Omega (39). One representative clone for each family was selected for periplasmic Nb expression, purification, and further characterization. To determine whether the clones from the four families of Nbs recognize CTNa_V_1.4–CaM or apo CaM, periplasmic-extract ELISA was performed. MaxiSorp plates were sensitized with 0.1 μg of either CTNa_V_1.4T–CaM, Ca^2+^ CaM, or apoCaM for 2 h at RT and then blocked with 5% bovine serum albumin (BSA) for 2 h at RT. After removal of blocking solution, 50 μl of a 1:100 dilution of TG1 periplasmic extracts expressing each Nb (Nb17, Nb26, Nb30, and Nb82) were incubated overnight at 4 °C (30). Nbs were detected using anti-HA-tag high-affinity secondary Ab by ELISA developed using an antirat IgG peroxidase–conjugated Ab. Nb17, Nb30, and Nb82 were then subcloned in pHEN6 vector. The coding regions were amplified by PCR using the following specific primers: forward (A6E): 5′-GATGTGCAGCTGCAGGAGTCTGGRGGAGG-3’;reverse (p38): 5′-GGACTAGTG CGGCCGCTGGAGACGGTGACCTGGGT-3’. The amplicons were purified from agarose gels, digested with restriction enzymes PsTI and BstEII, and cloned into the pHEN6-TEV-His vector for periplasmic expression of recombinant Nbs in *E. coli* Rosetta-gami 2 (DE3) cells. Nb82-His cloned into pHEN6-TEV-His was subcloned to generate Nb82-His-StrepII.

#### Purification of Nbs Nb17 and Nb82

The expression and purification of the Nbs was performed as described previously with minor modifications (30). Transformed *E. coli* Rosetta-gami 2 (DE3) cells were grown in LB medium supplemented with 100 μg/ml of carbenicillin, 50 μg/ml of kanamycin, and 20 μg/ml of chloramphenicol in addition to 1 mM MgCl_2_ and 0.1% glucose. Proteins were expressed by inducing the cultures with 1 mM IPTG at an absorbance of 0.8 to 0.9 at 600 nm at 18 °C at 200 rpm overnight. Cells were harvested by centrifugation and frozen at −80 °C for at least 2 h before proceeding to thawing and osmotic shock to extract the periplasmic proteins. For this, cells were thawed in a water bath, resuspended in osmotic-shock TES (0.2 M Tris–HCl, 0.65 mM EDTA, and 0.5 M sucrose, pH 8.0) buffer, and incubated at 4 °C for 1 h on an orbital shaker. Then, cells were further diluted with osmotic-shock TES buffer and incubated at 4 °C for 45 min on an orbital shaker. The periplasmic fraction was isolated by centrifugation at 8000 rpm at 4 °C for 30 min using an SLA1500 rotor. To the filtered supernatant (0.22 μm polyethersulfone filter), 2.5 mM Tris(2-carboxyethyl)phosphine and 5 mM imidazole were added and incubated overnight with 1 ml of prewashed Ni–NTA agarose Superflow beads at 4 °C using an orbital shaker. The Ni–NTA agarose beads were equilibrated with 50 mM Tris, 150 mM NaCl, pH 8.0 (TN buffer). Beads were washed in a gravity flow column using 40 ml of TN buffer (pH 8.0) with 10 mM imidazole, and Nbs were eluted using 40 ml of TN buffer (pH 8.0) with 300 mM imidazole. Purification fractions were loaded on an any kilodalton gel and bands analyzed by SDS-PAGE, and the Nb-containing elution fractions were pooled, dialyzed at 4 °C into low-salt TN buffer (20 mM Tris–HCl, 50 mM NaCl, 2 mM DTT, pH 7.5), and concentrated to 1 mg/ml before loading on a Superdex 75 column using TN buffer. The peak fractions from gel filtration containing the Nbs were then concentrated using a 3.5 kDa cutoff filtering units to a final concentration of ∼10 to 11 mg/ml as 24 μl aliquots and flash frozen and stored at −80 °C.

#### **Purification of CT regions of Na**_**V**_**1.4, Na**_**V**_**1.5, Na**_**V**_**1.7, and Na**_**V**_**1.9 in complex with CaM (CTNa**_**V**_**1.X–CaM)**

CT regions of Na_V_1.4(UniProtKb:P35499), Na_V_1.5 (UniProtKb:Q14524), Na_V_1.7 (UniProtKb:Q15858), and Na_V_1.9 (UniProtKb:Q9UI33) in complex with CaM (UniProtKb:P0DP23) (CTNa_V_1.X–CaM) were used. The constructs of the CT regions of the voltage-gated sodium channel isoforms were named consistently as “truncated (T)” for constructs ending at equivalent residue of Na_V_1.4_1764_ and “FL” for constructs ending at equivalent residue of Na_V_1.4_1836_. The constructs are CTNa_V_1.4T with amino acids 1599 to 1764, CTNa_V_1.4FL with amino acids 1599 to 1836, CTNa_V_1.5T with amino acids 1775 to 1940, CTNa_V_1.5FL with amino acids 1775 to 2016, CTNa_V_1.7T with amino acids 1761 to 1928, CTNa_V_1.7FL with amino acids 1761 to 1988, CTNa_V_1.9T with mino acids 1605 to 1768, and CTNa_V_1.9FL with amino acids 1605 to 1791 (Table S1 and Fig. 3*B*). Each construct was coexpressed with mammalian CaM and purified from BL21-CodonPlus RIL cells using a GST-sepharose column followed by anion exchange chromatography and a final gel filtration chromatography step as described by Yoder *et al.* (29) with minor modifications. In brief, cells were grown overnight at 37 °C in 100 ml of LB medium supplemented with 50 μg/ml kanamycin, 20 μg/ml chloramphenicol, and 100 μg/ml carbenicillin. About 10 ml of the overnight culture was used to inoculate 1 l of LB media containing the same antibiotics. The cells were grown at 37 °C to an absorbance of 0.8 to 0.9 at 600 nm, and protein expression was induced with 1 mM IPTG. The cells were grown overnight at 18 °C (approximately 18 h), centrifuged, and the cell pellet was frozen at −80 °C. After thawing, pellets were resuspended with PBS at 5× volume/weight ml/g of cells. DNAse was added, the cells were lysed using a microfluidizer, and the lysate was clarified at 27,500*g*. The supernatant was loaded on to a 3 ml GST resin using gravity flow. The column was washed with 30 ml wash buffer (PBS added with 100 mM NaCl), and free CaM was purified from this fraction for other experiments. The CTNa_V_T–CaM and CTNa_V_FL–CaM complexes were eluted in aliquots of 5 ml with an elution buffer containing 10 mM reduced l-glutathione in 50 mM Tris–HCl (pH 8.0). Eluted fractions containing protein were pooled, and 5 μg of PreScission protease was added per milligram of CTNa_V_–CaM for cleaving the GST tag. Dialysis was performed against 2 l of buffer containing 20 mM Tris, 50 mM NaCl, 1 mM DTT, and pH 7.4. The buffer was changed twice, and the final dialysis was allowed to proceed overnight at 4 °C. The dialyzed and PreScission protease-cleaved protein was loaded on a 15 ml Source Q anion exchange column (GE). Elution was performed using buffer 20 mM Tris, 1 mM DTT, pH 7.4 and a gradient of 50 to 500 mM NaCl. Free and cleaved GST eluted at ∼8 mS/cm, and CTNa_V_–CaM complexes eluted at between 14 and 27 mS/cm conductance that varied depending on the Na_V_ isoform. Fractions were judged to be >95% pure by SDS-PAGE gel and then pooled and concentrated to ∼15 to 20 mg/ml and flash frozen and stored at −80 °C. In the case of CTNa_V_1.9T and CTNa_V_1.9FL, CaM did not coelute with the CTNa_V_s unlike the other cases.

#### **Detection of Nb specificity for Na**_**V**_**s by ELISA**

Purified Nbs were assessed for recognition of Na_V_ proteins in high-binding 96-well plates. The analytes, purified CTNa_V_T–CaM and CTNa_V_FL–CaM protein isoforms (CTNa_V_1.4T–CaM, CTNa_V_1.4FL–CaM, CTNa_V_1.5T–CaM, CTNa_V_1.5FL–CaM, CTNa_V_1.7T–CaM, and CTNa_V_1.7FL–CaM), CTNa_V_1.9T, CTNa_V_1.9FL, and CaM, were diluted in PBS and coated (1 μg/well) to a 96-well plate using carbonate–bicarbonate buffer, pH 9.5, at 4 °C overnight. Next, the plate was washed five times using 100 μl/well wash buffer (PBS added with 0.1% Tween-20). Then, protein-coated wells were incubated with 100 μl/well of 0.01 μg of Nb17 or Nb82 diluted in 1× blocking buffer (PBS added with 2% BSA) for 2 h at RT on a shaking platform. The plates were then washed five times using 100 μl/well of wash buffer and incubated with mouse anti-His-peroxidase Ab at 100 mU/ml in blocking buffer for 1 h at RT. The plates were finally washed in wash buffer five times, and peroxidase activity was assayed using 100 μl/well o-phenyl diamine in 150 mM citrate phosphate buffer and 30% H_2_O_2_. The plates were incubated in the dark for a few minutes until a yellow color developed in at least one of the wells. The reaction was stopped by the addition of 100 μl/well of 2 N H_2_SO_4_, and the absorbance was read at 450 nm (20 flashes) using a microplate reader.

#### DSC experiments

DSC experiments were performed using a MicroCal capillary DSC instrument. Purified Nbs, Nb17 and Nb82, were dialyzed into buffer containing 10 mM PBS, pH 8.0, with 1 mM Tris(2-carboxyethyl)phosphine, and further diluted with the dialysate to an experimental concentration of ∼0.15 mg/ml. The exact concentration of each protein solution was determined from the absorbance at 280 nm. Each solution was thoroughly degassed before loading of the calorimetric cell to have an effective volume of 138 μl. The reference cell was loaded with the dialysate. Thermal scans were conducted from 10 to 90 °C at a rate of 1 °C/min. The raw data were collected and processed using the software provided with the instrument. Data were analyzed after baselines were subtracted as described by Freire (40). The resulting transition excess heat capacity curves were fitted by nonlinear least squares in terms of reversible or irreversible denaturation models depending on whether the transitions were reversible or irreversible (33). For reversible transitions, the fitting procedure yields the calorimetric enthalpy, van’t Hoff enthalpy, and transition temperature. For irreversible transitions, the fitting procedure yields the calorimetric enthalpy, activation energy, and transition temperature.

#### Crystallization of Nb82

Purified Nb82 was used at 10 mg/ml for all crystallization experiments. Sparse matrix commercial crystallization screens were used to find conditions in hanging-drop vapor diffusion by mixing equal volumes of Nb82 and reservoir solution. Nb82 crystallized in 2% (w/v) PEG monomethyl ether 550, 1.8 M ammonium sulfate, 0.1 M Bis–Tris, pH 6.5 was used for X-ray diffraction experiments. Crystals appeared as needles after 1 day of equilibration at 20 °C and reached 100 μm in their longest dimension on day 30. These needles were used to macro seed into 2 μl hanging-drop vapor diffusion plates with drops containing equal volumes of 10 mg/ml Nb82 and reservoir conditions optimized around the original crystallization condition varying the concentrations of PEG and ammonium sulfate. New crystals appeared in 2% (w/v) PEG monomethyl ether 550, 1.5 to 1.8 M ammonium sulfate, 0.1 M Bis–Tris, pH 6.5, on day 5. The crystals grew into cubes with largest samples being 125 μm in their longest dimension and were harvested on day 30 postseeding from the mother liquor mixed with 1 M lithium sulfate as the cryoprotectant into Hampton loops and plunge-frozen into liquid nitrogen.

#### Data collection and structure refinement

X-ray diffraction data of the Nb82 crystal were collected at 100 K at the NSLS II 17-ID-1 (AMX) on a DECTRIS Eiger 9M. Data were processed with fastdp (41) and XDS (42) and scaled (42) using XSCALE. Initial phases were obtained by molecular replacement using an Nb structure as search model (PDB ID: 5LMJ (14)) with the CCP4 program PHASER (43). Initial models were improved with multiple rounds of rebuilding using Coot (44) and refinement using REFMAC, version 5.8 (45). The quality of the model was assessed with Coot (44) validation tools and the wwPDB validation servers (46). Statistics are shown in Table 1. The final model contains four Nb82 molecules in the asymmetric unit.

#### **Nb-mediated shift of Na**_**V**_**s by SEC**

Mobility in-gel filtration chromatography was performed to verify formation of CTNa_V_–CaM + Nb complexes using purified proteins (Figs. 5 and 6). Nbs and Na_V_s were mixed in a 1.2:1 M ratio, incubated for 2 h to overnight at 4 °C, and run on a Superdex 75 column using 20 mM Tris, 50 mM NaCl, and pH 7.4. Chromatograms were exported as Excel files to GraphPad Prism (GraphPad Software, Inc) for analysis and plotting. About 0.5 mg of egg-white lysozyme (14 kDa) and 0.5 mg of BSA (66 kDa) were run to serve as molecular weight gel filtration standards using the same experimental conditions to evaluate the molecular weight of the peak elution fractions.

#### Nb17 and Nb82 binding kinetics using BLI

Nb17 and Nb82 binding to CTNa_V_1.4 and CTNa_V_1.5 was measured by BLI. Data were acquired using the Data acquisition software, version 11.0, and analyzed using the Data analysis software, version 11.0 (ForteBio, Sartorius Corp). The assay was performed in kinetics mode using 200 μl protein/well in a 96-well plate format. His-tagged proteins, Nb17 and Nb82, or just buffer was immobilized on Ni–NTA biosensors. Nb17 and Nb82 loading was done at 2.5 μg/ml concentration for 300 s to prevent overcrowding and self-association of the ligand. CTNa_V_1.4T–CaM, CTNa_V_1.5T–CaM, CTNa_V_1.7T–CaM, and CTNa_V_1.9T–CaM were tested as analytes. To measure Nb association with Na_V_ proteins, the Nb-loaded Ni–NTA sensors were first transferred to wells containing blocking reagent (0.1% biocytin) in assay buffer for 150 s to prevent nonspecific binding and then to wells containing assay buffer until a stable baseline was reached (100 s). Following this, sensors were dipped into wells with 1:2 serially diluted Na_V_ proteins (at 200, 100, 50, 25, 12.5, and 6.25 nM concentrations) for 300 s followed by a 300 s dissociation step in assay buffer. All experiments were carried out at 25 °C and acquisition standard kinetics at 5 Hz with the assay plate shaking at 1000 rpm.

#### Flow cytometric FRET two-hybrid assay

Flow cytometric FRET two-hybrid assay for detecting Nb interaction with Na_V_1.5 was performed as in previous studies (34). Briefly, HEK293 cells were cultured in 12-well plates and transfected with PEI 25 kDa linear polymer (Polysciences; catalog no.: 2396602). For each experiment, we cotransfected: 0.5 μg of Nb17 or Nb82 fused to Cerulean; 2 μg of Venus-tagged FL Na_V_1.5; and 0.5 μg of t-Antigen. To test the interaction of Na_V_1.x isoforms to the Nbs, we cotransfected 0.5 μg of Nb17 or Nb82 fused to Cerulean, 2 μg of Venus-tagged CTNa_V_1.x; and 0.5 μg of t-Antigen. The cDNA pairs were mixed together in 200 μl of serum-free Dulbecco's modified Eagle's medium (DMEM) media, and 5 μl of PEI was added into each sterile tube. Following 15 min of incubation, PEI–cDNA mixtures were added to the 12-well plates, and cells were cultured for 2 days prior to experimentation. Protein synthesis inhibitor, cycloheximide (100 μM), was added to cells 2 h prior to experimentation to enhance fluorophore maturation. For FRET measurements, we utilized an LSR II (BD Biosciences) flow cytometer equipped with 405, 488, and 633 nm lasers for excitation and 18 different emission channels as previously described (34). Forward and side scatter signals were detected and used to gate for single and healthy cells. Fluorescence emission from three different channels (BV421, FITC, and BV510) were used to estimate fluorescence emission in the donor, acceptor, and FRET channels. Data were analyzed using custom MATLAB software (Mathworks).

#### **Western blots using Nb82 to detect purified CTNa**_**V**_**–CaM proteins**

Western blot analysis was performed to assess whether Nb82 can detect CTNa_V_1.4 and CTNa_V_1.5 proteins using Nb82 as the primary Ab. For this, ∼1 μg/lane of purified CTNa_V_1.4T–CaM, CTNa_V_1.4FL–CaM, CTNa_V_1.5T–CaM, CTNa_V_1.5FL–CaM, CTNa_V_1.7T–CaM, CTNa_V_1.7FL–CaM, CTNa_V_1.9T, CTNa_V_1.9FL, CaM, Nb82-His, Nb17-His proteins were run on an Any kD Mini-PROTEAN TGX gel at 180 V for 30 min. Proteins were transferred using the P3 program (20 V for 10 min) of the iBlot2 protein transfer system and blocked with blocking buffer (1× PBS with Tween-20 [PBST], pH 7.4 with 5% nonfat dry milk) for 27 °C for 1 h and then washed five times in PBST (0.1% Tween-20). Proteins were incubated with Nb82-His (1 μg/ml) in blocking buffer shaking in the cold room overnight. Blots were further washed five times in PBST and probed using an anti-His-HRP-Ab at 1:500 dilutions in blocking buffer by incubating at 27 °C for 1 h with shaking. Western blot was developed and visualized using the GeneSys image acquisition software to obtain exposures at 20 s, 2 min, and 5 min. The blots were exported as tiff images and viewed and edited using ImageStudioLite (Li-COR Biosciences).

#### hiPSC differentiation

HiPSCs were reprogrammed from peripheral blood mononuclear cells of a healthy donor and differentiated into ventricular-like CMs by temporal modulation of Wnt signaling. Cells were cultured in basal media (RPMI1640 with 2.5 mM glutamine Gibco; catalog no.: 11875093) with B27[−] supplement without insulin (Gibco; catalog no.: 10889038) and treated with small-molecule CHIR99021 (Tocris, R&D Systems; catalog no.: 4423) on differentiation day 0 (dd0) and IWR-1 (Sigma; catalog no.: I0161) on dd3. hiPSC–CMs were replated and switched to B27(+) supplement with insulin (Gibco; catalog no.: 17504044) once they began beating ∼dd9. hiPSC–CMs were purified with lactate media from dd16 to dd20 (basal media DMEM without glucose or pyruvate [Gibco; catalog no.: 11966025]; 50 mM Hepes buffer; 100× GlutaMAX [Gibco; catalog no.: 35050061]; 100× minimum essential medium nonessential amino acids [Gibco; catalog no.: 11140050); and 4 mM sodium l-lactate [Sigma; catalog no.: L7022]). hiPSC–CMs formed an autorhythmic contracting sheet of cells and were pelleted in cold PBS on dd36 for analysis by Western blot.

#### **Western blots using Nb82 to detect endogenous Na**_**V**_**1.4 and Na**_**V**_**1.5**

Western blot analysis was performed to assess whether Nb82 could detect Na_V_1.4 in skeletal muscle homogenates and Nav1.5 in heart of adult mouse and also Nav1.5 in cell lysate from hiPSC–CM. Heart muscle, brain, and quadriceps femoris muscle were collected from a 16-week-old female C57BL/6 mouse and snap frozen before analysis by Western blot. Tissue samples were crushed using a tissue-grinder pestle and resuspended in 1× PBS (pH 7.4) with 500 mM NaCl and protease inhibitors and sonicated in the cold room with a 10 s pulse–20 s pause program for a total of 10 min. Following sonication, 2% (v/v) Triton X-100 was added to the lysate and incubated on ice for 30 min. The lysate was then centrifuged in a table-top centrifuge at 10,000*g* at 4 °C for 30 min. The supernatants were mixed with equal volumes of 2× SDS-loading buffer and loaded at 40 μl/lane and run on an NuPAGE 4 to 12% Bis–Tris gel using 1× MES–SDS running buffer (Novagen) at 230 V for 72 min. Proteins were transferred, and blot was developed as described previously using blocking buffer (1× PBST [pH 7.4] with BSA). The blots were probed with Nb82 followed by anti-His-HRP detection or by pan-Na_V_ Ab and antimouse IgG-HRP or using anti-His-HRP Ab alone as control.

### Materials and reagents

#### CTNa_V_–CaM proteins

Genes coding for CT region of human Na_V_ (CTNa_V_) isoforms, namely, CTNa_V_1.4T (amino acids 1599 to 1764), CTNa_V_1.4FL (amino acids 1599 to 1836), CTNa_V_1.5T (amino acids 1775 to 1940), CTNa_V_1.5FL (amino acids 1775 to 2016), CTNa_V_1.7T (amino acids 1761 to 1928), CTNa_V_1.7FL (amino acids 1761 to 1988), CTNa_V_1.9T (amino acids 1605 to 1768), and CTNa_V_1.9FL (amino acids 1605 to 1791), were subcloned into pGEX 6p1 expression vector (GenScript) and coexpressed with mammalian CaM in BL21-CodonPlus RIL *E. coli* cells (Agilent), purified using GST-sepharose 4b resin (GE Lifesciences), ReSource Q anion exchange column (GE, MilliporeSigma).

#### Llama immunization and Nb phage library

Freund’s adjuvant (Sigma), ELISA of immune sera using MaxiSorp plates (Thermo Scientific), PBL cells isolated using Ficoll–Paque (GE Healthcare), total RNA isolation using RNeasy Midi Kit (Qiagen), restriction enzymes for cDNA amplification, PstI and NotI (New England Biolabs), *E. coli* TG1 cells (Lucigen) for phage display library, plasmid DNA isolation using QIAprep Spin Miniprep kit (QIAGEN), trypsin (Gibco), panning in deep-well plates (Greiner Bio-One), phage ELISA primary Ab anti-HA high affinity, Roche and secondary Ab antirat IgG peroxidase-conjugate (Sigma), BSA (Sigma), BstEII (New England Biolabs). Genes coding for Nb17 and Nb82 were subcloned into pHEN6-TEV-His vector (GenScript), and Nb82 was also subcloned into pHEN6-TEV-His-StreptagII vector (GenScript); *E. coli* Rosetta-gami 2 (DE3) cells (Novagen).

#### Nb purification

His-Pur Ni–NTA superflow agarose (Thermo Scientific), Superdex 75 (10/300) gel filtration column (MilliporeSigma, Sigma–Aldrich), any-kDa gel 10-15well (Mini-PROTEAN TGX Precast gel; Bio-Rad) 3.5 kDa cutoff concentrators (polyethersulfone membrane; MilliporeSigma).

#### Protein ELISAs and Western blots

About 96-well EIA/RIA plates (Costar-9018), anti-His-peroxidase secondary Ab (Roche), Tecan infinite M1000 microplate reader (Tecan i-control; 2.0.10.0 application), iBlot2 transfer stack (Thermo Scientific; catalog no.: IB24001), iBlot2 protein transfer system (Thermo Scientific), anti-His-HRP-Ab (Sigma; catalog no.: 11965085001), pan-Na_V_ Ab (Sigma; catalog no.: S8809), 1 μg/ml of antimouse IgG-HRP-Ab (Cell Signaling; catalog no.: 7076S), and Supersignal West Pico chemiluminescent substrate (Thermo Fisher Scientific; catalog no.: 34080).

#### Cell culture

HEK293 cells (American Type Culture Collection; catalog no.: CRL-1573), DMEM with 4.5 g/l d-glucose, 110 mg/l sodium pyruvate, 2 mM l-glutamine (Gibco; catalog no.: 11995040), additional 4 mM l-glutamine (Gibco; catalog no.: 35650061), fetal bovine serum (Sigma; catalog no.: F2442), and PEI 25 kDa linear polymer (Polysciences; catalog no.: 2396602). Human Na_V_1.5 construct corresponds to clone M77235.1 (GenBank). Reagents and small molecules for hiPSC culture and differentiation: basal media (RPMI 1640 with 2.5 mM glutamine; Gibco; catalog no.: 11875093), B27(−) supplement without insulin (Gibco; catalog no.: 10889038), CHIR99021 (Tocris, R&D Systems; catalog no.: 4423), IWR-1 (Sigma; catalog no.: I0161), and B27(+) supplement with insulin (Gibco; catalog no.: 17504044). Lactate purification media: (basal media DMEM without glucose or pyruvate (Gibco; catalog no.: 11966025); 50 mM Hepes buffer; 100× GlutaMAX (Gibco; catalog no.: 35050061); 100× minimum essential medium nonessential amino acids (Gibco; catalog no.: 11140050); 4 mM sodium l-lactate (Sigma; catalog no.: L7022).

#### CTNaV1.x constructs for flow cytometric FRET two-hybrid assay

Venus coding sequence followed to the coding sequence for a triple alanine and FL Na_V_1.5 was cloned in pcDNA3.1. The Venus-AAA-CTNa_V_1.x contains the coding sequence for CTNa_V_1.1 (amino acids 1788 to 2009), CTNa_V_1.2 (amino acids 1775 to 2005), CTNa_V_1.3 (amino acids 1770 to 2000), CTNa_V_1.4 (amino acids 1598 to 1835), CTNa_V_1.5 (amino acids 1771 to 2016), CTNa_V_1.6 (amino acids 1765 to 1980), CTNa_V_1.7 (amino acids 1759 to 1988), CTNa_V_1.8 (amino acids 1721 to 1956), and CTNa_V_1.9 (amino acids 1603 to 1791).

### Equipment/apparatus

Octet RED96e instrument (ForteBio, Sartorius Corp), MicroCal capillary cell Differential Scanning Calorimeter (Malvern Panalytical), Akta purification system, SLA1500 rotor (Thermo Fisher Scientific), microfluidizer (Microfluidics Corporation), Q125 sonicator (QSonica), Mosquito for 96-well plate screens (SPT Labtech), NSLS II 17-ID-1 beamline equipped with a DECTRIS Eiger 9M detector, LSR II Flow cytometer for FRET two-hybrid assay (BD Biosciences).

## Data availability

The structure presented in this article has been deposited in the PDB with PDB ID: 7R63. All clones and constructs will be shared upon request to gabelli@jhmi.edu. All remaining data are contained within this article and the supporting information.

## Supporting information

This article contains supporting information (14, 29, 30, 41, 42, 43, 44, 45, 46).

## Conflict of interest

S. B. G. is a founder and holds equity in Advanced Molecular Sciences LLC. S. B. G. is consultant to Genesis Therapeutics and Xinthera, Inc. All other authors declare that they have no conflicts of interest with the contents of this article.
