# Supplementary material for: Development of high-affinity nanobodies specific for NaV1.4 and NaV1.5 voltage-gated sodium channel isoforms
Source: J Biol Chem. 2022 Feb 21;298(4):101763. doi: 10.1016/j.jbc.2022.101763 (PMC8935509; doi:10.1016/j.jbc.2022.101763)
Supplement: Supplementary Figures S1–S3 and Tables S1–S2 [file mmc1.docx]

Development of High affinity Nanobodies Specific for Na_V_1.4 and Na_V_1.5 Voltage-Gated Sodium Channel Isoforms

Lakshmi Srinivasan^1^, Vanina Alzogaray^2^, Dakshnamurthy Selvakumar^3^, Sara Nathan^1^, Jesse B. Yoder^1, #^, Katharine M. Wright^1^, Sebastián Klinke^2^, Justin N. Nwafor^1^, María S. Labanda^2^, Fernando A. Goldbaum^2^, Arne Schön^5^, Ernesto Freire^5^, Gordon F. Tomaselli^6^, L. Mario Amzel^1^, Manu Ben-Johny^4^, Sandra B. Gabelli^1,6,7^

SUPPORTING INFORMATION

Supporting Figures and tables

**Supporting Figures**

**A.**





**B.**


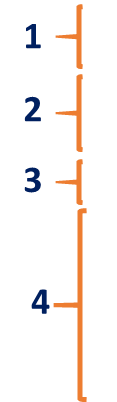

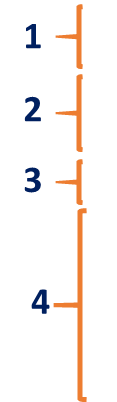

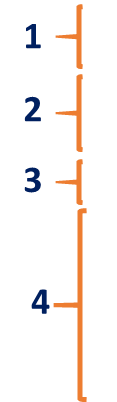

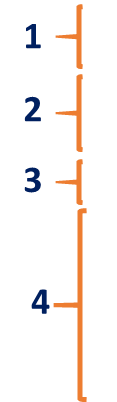

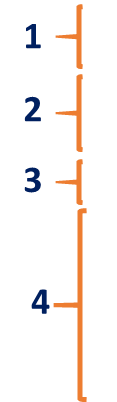

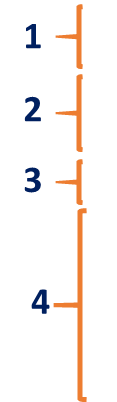


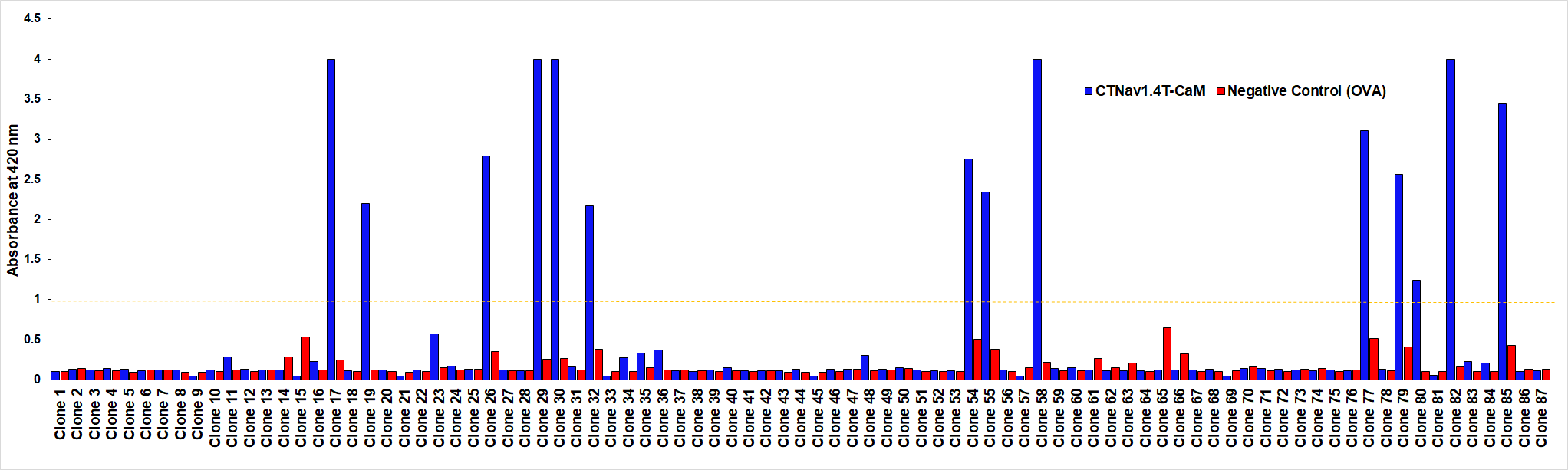


**C.**

**Fig. S*1*. Nanobody clones selected by phage display library panning.***A*, ELISA of immune serum from llama at Day 35 following immunization with CTNa_V_1.4T-CaM (aa 1599-1764). The graphed absorbance was subtracted from the control value without antigen depending on the dilution of serum. *B*, ELISA of 87 clones after the two rounds of panning. 14 different anti-Na_V_1.4 Nbs were identified using CTNa_V_1.4T-CaM as bait. Absorbance values greater than 1 (orange dashed line) were considered positive. *C*, Sequence alignment of 14 llama-derived anti-CTNa_V_1.4 Nbs classified into 4 unique Nb families (1-4). Family 1 (Nb17 and 29), Family 2 (Nb82, 19 and 80), Family 3 (Nb30 and 58), Family 4 (Nb26, 54, 55, 77, 79, 32 and 85). The three CDR regions are color coded as CDR1 (blue), CDR2 (green), and CDR3 (magenta).

7R63 (Nb82)

**A.**

**B.**


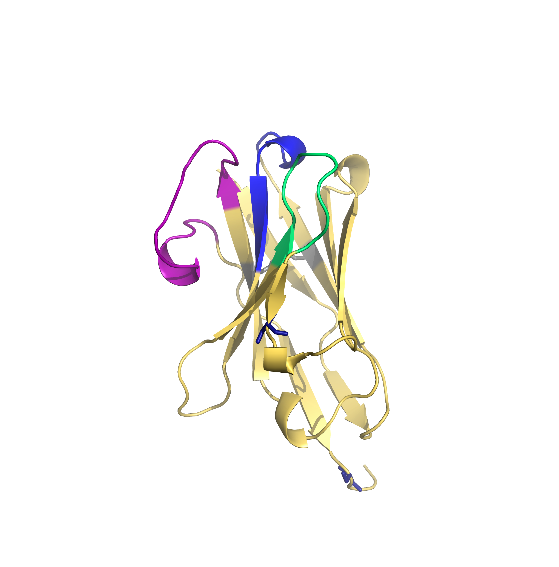


**Fig. S*2*. Structural alignment of llama Nbs highlights the diversity in the CDR3 fold.** *A*, Structure of Nbs displaying the CDR3 regions (boxed) of, PDB IDs 7R63 (Nb82) (yellow), 5LMJ (blue), 6H6H (grey), and 5LZ0 (purple). Nb82 orientation and color coded same as in Fig. 3*C and D*. *B*, Sequence alignment of Nb82 and other nanobodies shown in A displaying divergent CDR regions, CDR1 (blue), CDR 2 (green), and CDR3 (magenta). Identical amino acids are in white with background in orange, similar amino acids are in orange with white background, different amino acids are in black with white background.







**D.**

**A.**

**C.**

**B.**

**Figure S*3*. Nb17 forms a stable complex with CTNa_V_1.5-CaM.** A. Size exclusion chromatography profile for CTNa_V_1.5T-CaM+Nb17 (solid green line) compared with CTNa_V_1.5T-CaM (dashed green line). The Nb17 elution profile is shown in magenta. B. SDS-PAGE gel of the fractions from A. C. Size exclusion chromatography profile for CTNa_V_1.5FL-CaM+Nb17 (solid green line, 2 peaks) compared with CTNa_V_1.5FL-CaM (dashed green line). The Nb17 elution profile is shown in magenta. D. SDS-PAGE gel of the elution fractions from C. Gel filtration molecular weight standards; BSA (66 kDa) (dashed gray line) and lysozyme (14 kDa, solid gray line) are included in both panels A and C.

**Supporting Tables**

Table S*1*. Pairwise sequence identities of 1-2 linker and CT regions of Nav1.4 to Nav1.5, Nav1.7 and Nav1.9

|  | CTNav1.5 | CTNav1.4 | CTNav1.7 | CTNav1.9 |
| --- | --- | --- | --- | --- |
| 1-2 linker -Nav1.4 | Range 1: Identities: 46%, Positives: 65%  Gaps: 0%  Range 2:  Identities: 61% Positives: 76%  Gaps: 0% | Identities: 100 %  Positives: 100 %  Gaps: 0 % | Range 1:  Identities: 63% Positives: 78%  Gaps: 0%  Range 2:  Identities: 67% Positives: 81%  Gaps: 0% | Identities: 29%  Positives: 40%  Gaps: 27% |
| CTNav1.4 | Identities: 74% Positives: 85%  Gaps: 2% | Identities: 100 %  Positives:100 %  Gaps: 0 % | Identities: 65%  Positives: 76%  Gaps:4% | Identities: 64%  Positives: 78%  Gaps: 0% |

Table S*2*. Amino acids included in the CTNa_V_T and CTNa_V_FL constructs

| CTNa_V_  constructs | residues included in the constructs |
| --- | --- |
| CTNa_V_1.4T | 1599-1764 |
| CTNa_V_1.4FL | 1599-1836 |
| CTNa_V_1.5T | 1775-1940 |
| CTNa_V_1.5FL | 1775-2016 |
| CTNa_V_1.7T | 1761-1928 |
| CTNa_V_1.7FL | 1761-1988 |
| CTNa_V_1.9T | 1605-1768 |
| CTNa_V_1.9FL | 1605-1791 |

REFERENCES

1. Pardon, E.; Laeremans, T.; Triest, S.; Rasmussen, S. G.; Wohlkonig, A.; Ruf, A.; Muyldermans, S.; Hol, W. G.; Kobilka, B. K.; Steyaert, J., A general protocol for the generation of Nanobodies for structural biology. *Nat Protoc* **2014,** *9* (3), 674-93.

2. Yoder, J. B.; Ben-Johny, M.; Farinelli, F.; Srinivasan, L.; Shoemaker, S. R.; Tomaselli, G. F.; Gabelli, S. B.; Amzel, L. M., Ca(2+)-dependent regulation of sodium channels Na_V_1.4 and Na_V_1.5 is controlled by the post-IQ motif. *Nature communications* **2019,** *10* (1), 1514.

3. Winter, G.; McAuley, K. E., Automated data collection for macromolecular crystallography. *Methods* **2011,** *55* (1), 81-93.

4. Kabsch, W., XDS: Integration, scaling, space-group assigment and post refinement. *Acta Crystallogr D Biol Crystallogr* **2010,** *66* (Pt 2), 125-32.

5. Duhoo, Y.; Roche, J.; Trinh, T. T. N.; Desmyter, A.; Gaubert, A.; Kellenberger, C.; Cambillau, C.; Roussel, A.; Leone, P., Camelid nanobodies used as crystallization chaperones for different constructs of PorM, a component of the type IX secretion system from Porphyromonas gingivalis. *Acta Crystallogr F Struct Biol Commun* **2017,** *73* (Pt 5), 286-293.

6. McCoy, A. J.; Grosse-Kunstleve, R. W.; Adams, P. D.; Winn, M. D.; Storoni, L. C.; Read, R. J., Phaser crystallographic software. *J Appl Crystallogr* **2007,** *40* (Pt 4), 658-674.

7. Emsley, P.; Lohkamp, B.; Scott, W. G.; Cowtan, K., Features and development of Coot. *Acta Crystallogr D Biol Crystallogr* **2010,** *66* (Pt 4), 486-501.

8. Collaborative Computational Project, N., The CCP4 suite: programs for protein crystallography. *Acta Crystallogr D Biol Crystallogr* **1994,** *50* (Pt 5), 760-3.

9. Berman, H.; Henrick, K.; Nakamura, H., Announcing the worldwide Protein Data Bank. *Nat Struct Biol* **2003,** *10* (12), 980.
